# Carotenoids in orange carrots mitigate non-alcoholic fatty liver disease progression

**DOI:** 10.3389/fnut.2022.987103

**Published:** 2022-09-26

**Authors:** Emilio Balbuena, Junrui Cheng, Abdulkerim Eroglu

**Affiliations:** ^1^Plants for Human Health Institute, North Carolina State University, Kannapolis, NC, United States; ^2^Department of Molecular and Structural Biochemistry, College of Agriculture and Life Sciences, North Carolina State University, Raleigh, NC, United States

**Keywords:** phytochemicals, nutrition, beta-oxidation, nuclear receptors, lipid metabolism

## Abstract

**Background:**

Carotenoids are abundant in colored fruits and vegetables. Non-alcoholic fatty liver disease (NAFLD) is a global burden and risk factor for end-stage hepatic diseases. This study aims to compare the anti-NAFLD efficacy between carotenoid-rich and carotenoid-deficient vegetables.

**Materials and methods:**

Male C57BL/6J mice were randomized to one of four experimental diets for 15 weeks (*n* = 12 animals/group): Low-fat diet (LFD, 10% calories from fat), high-fat diet (HFD, 60% calories from fat), HFD with 20% white carrot powders (HFD + WC), or with 20% orange carrot powders (HFD + OC).

**Results:**

We observed that carotenoids in the orange carrots reduced HFD-induced weight gain, better than white carrots. Histological and triglyceride (TG) analyses revealed significantly decreased HFD-induced hepatic lipid deposition and TG content in the HFD + WC group, which was further reduced in the HFD + OC group. Western blot analysis demonstrated inconsistent changes of fatty acid synthesis-related proteins but significantly improved ACOX-1 and CPT-II, indicating that orange carrot carotenoids had the potential to inhibit NAFLD by improving β-oxidation. Further investigation showed significantly higher mRNA and protein levels of PPARα and its transcription factor activity.

**Conclusion:**

Carotenoid-rich foods may display more potent efficacy in mitigating NAFLD than those with low carotenoid levels.

## Introduction

Non-alcoholic fatty liver disease (NAFLD) is characterized by a 5–10% excessive lipid deposit in hepatocytes without significant alcohol intake (1). It is the most common form of chronic liver disease in the USA, affecting 80–100 million individuals (2). Obesity has been shown to be the principal risk factor for NAFLD development (3). Although isolated hepatic steatosis is considered a benign condition or minimal rate of progression (4), NAFLD patients generally have a higher risk of developing end-stage liver diseases and increased morbidity and all-cause mortality than healthy individuals (5, 6). Currently, there is no FDA-approved medication for NAFLD, and the optimal method of alleviating NAFLD is the adoption of lifestyle changes, including weight management and embracing healthy eating habits (7).

Carotenoids are naturally occurring pigments synthesized by plants, algae, and photosynthetic bacteria (8–10). They can scavenge reactive oxygen species and deactivate electronically excited sensitizer molecules (11). Accumulating evidence has shown health benefits of carotenoids in various organs (12–15). Recently, a randomized, double-blinded trial showed that compared to consuming vegetable pastes containing low levels of carotenoids, the consumption of carotenoid-rich vegetable pastes promoted the decrease of visceral fat and waist circumference among obese subjects (16), indicating that carotenoids from whole foods may display an independent beneficial efficacy against obesity. However, the role of fruits or vegetables against NAFLD was less evident. A cross-sectional study with middle-aged subjects failed to observe a significant association between fruit or vegetable intake and NAFLD (17). Consistently, a meta-analysis with six cross-sectional studies and two case-control studies found the association between fruit intake and the likelihood of developing NAFLD to be not beneficial (18). A potential explanation for the negative results might be the misclassification of the food groups by combining the low-carotenoid food with those enriched with carotenoids. Therefore, the major knowledge gap is whether whole foods containing high carotenoid levels (such as orange carrots) are more efficient in preventing NAFLD than low-carotenoid foods (such as white carrots).

The efficacy of β-carotene in ameliorating NAFLD has been shown in multiple studies (19–21). However, realizing that single nutrient theories were inadequate to explain the efficacy of daily dietary changes on non-communicable diseases (22), an increasing number of nutritional studies shifted their focus from single nutrients to whole foods. Specifically, scientists in the carotenoid studies found that whole food enriched with β-carotene, such as apricots and Campari tomatoes, were effective in mitigating diet-induced NAFLD (23, 24). Nevertheless, these foods contain a wide arrange of compounds such as fiber, so whether the anti-NAFLD efficacy was mainly from carotenoids or other compounds remains unknown. Another class of food-derived compounds known as flavonoids have been documented to provide health benefits through anti-hyperlipidemic, anti-inflammatory, and anti-diabetic effects against high-fat and high-fructose conditions (25).

From the mechanistic point of view, NAFLD results from imbalanced hepatic lipid homeostasis, which can be caused by increased hepatic triglyceride (TG) uptake, enhanced fatty acid synthesis, or decreased β-oxidation. Peroxisome proliferator-activated receptors (PPARs) belong to a ligand-activated transcription factor superfamily comprising three isoforms: alpha (α), beta/delta (β/δ), and gamma (γ) (26). PPARα is ubiquitously expressed while highly expressed in the liver (26, 27). Over the last several decades, various studies have focused on investigating PPARα due to its critical role in lipid and lipoprotein metabolism (28–30) by regulating a wide spectrum of target genes such as acyl-CoA oxidase 1 (ACOX1) (30) and carnitine palmitoyltransferase-I (CPT-I) (31). AMP-activated protein kinase (AMPK) is a master regulator of metabolism, which coordinates metabolic pathways and therefore maintains energy homeostasis (32). Interestingly, genetic liver-specific AMPK activation in mice alleviated the diet-induced obesity and NAFLD, suggesting that AMPK could be a potential target for preventing NAFLD (33). Since several publications reported that dietary carotenoids and their metabolites were effective in upregulating PPARα and activating AMPK (34–36), we performed further studies focusing on these targets.

To conclude, the major gap of knowledge in the carotenoid and NAFLD research relates to the failure of introducing a positive control that contain the similar compounds from the food source other than carotenoids. Therefore, the objective of this study was to compare the anti-NAFLD efficacy between carotenoid-rich vegetables (orange carrots) and carotenoid-deficient vegetables with similar concentrations of other compounds (white carrots). We hypothesize that orange carrots are more efficient in mitigating NAFLD in HFD-induced obese mice compared with white carrots.

## Materials and methods

### Animals

All animal protocols for the study were approved by the Institutional Animal Care and Use Committee (IACUC) at North Carolina State University. C57BL6J mice (male, *n* = 48) were purchased from Jackson Laboratory (Bar Harbor, ME, USA). Four mice were co-housed in one cage in a controlled temperature and humidity room with a 12-h light/dark cycle and fed with a standard chow diet. At 11 weeks of age, mice were randomized to four dietary groups (*n* = 12 in each group): low-fat diet (LFD, D12450J, 10% calories from fat), high-fat diet (HFD, D12492, 60% calories from fat), HFD with 20% w/w white carrot (HFD + WC), and HFD with 20% w/w orange carrot (HFD + OC). We utilized 20% (w/w) carrots due to the limited bioaccessibility of carotenoids (37). Moreover, the cleavage efficiency of carotenoids to produce apocarotenoids is much higher in mice than in humans (38, 39). Therefore, although 20% carrots seem to be a supraphysiological dose in human beings, such dosage serves the purpose of this study (our research interests focus on carotenoids, not carotenoid metabolites). Food and water were administered *ad libitum.* Compositions of dietary pellets and vitamin mix (V10001) have been provided in Supplementary Table 1. The dietary intervention lasted for 15 weeks. Carrots were purchased from local grocery stores and lyophilized at NC Food Innovation Lab in Kannapolis, NC. All diet pellets were made at Research Diets Inc. (New Brunswick, NJ, USA). Body weight for each mouse and the food consumption amount were recorded weekly. Submandibular blood collection was performed every month; one mouse in the LFD group was deceased during the submandibular blood collection, making LFD *n* = 11 for subsequent studies. At the end of the study, mice were anesthetized with isoflurane, followed by cardiac puncture. Blood was transferred to a capillary blood collection tube and placed at room temperature for 30 min. Serum was collected by centrifuging the blood at 1,500 x *g* for 10 min at 4°C. Livers were weighed and washed with saline. A piece of liver tissue was fixed in 10% formalin (Thermo Fisher Scientific, Waltham, MA, USA) for histopathological examination. All the tissues were harvested, snap-frozen in liquid nitrogen, then stored at −80°C for further analysis.

### Body composition analysis

EchoMRI-100 (EchoMRI, Houston, TX, USA) was used to measure body composition measurements of fat and lean masses in mice at baseline and the end of the study. Briefly, non-anesthetized mice were placed in a restraining cylinder, locked using the Velcro attachment. The tube was subsequently inserted to the chamber unit of the equipment, and mice’s fat mass, lean mass, free water, and total water mass were determined.

### Liver and serum triglyceride analysis

Liver and serum TG levels were assessed using a commercialized colorimetric kit (Abcam, Waltham, MA, USA). Weighed liver samples (approximately 100 mg) were homogenized in an extraction buffer (5% Tween-20 in deionized water). Serum samples were 1:1 diluted with the extraction buffer. Both liver and serum mixtures were incubated at 90°C for 3 min and centrifuged at 24,532 x *g* for 2 min. Then the supernatants, lipase, TG probe and TG enzyme mix were successively added to the 96-well plate according to the manufacturer’s protocol. The output was measured on a microplate reader at OD 570 nm.

### Histology

Liver tissues were fixed in 10% formalin and washed with 70% ethanol. Then, the livers were placed in a tissue processor for dehydration, clearing, and paraffin wax infiltration, followed by paraffin embedding and sectioning. Five-micrometer sections of liver tissues were stained with hematoxylin and eosin and examined using a ZEISS Axio Observer microscopy platform (Carl Zeiss Microscopy, White Plains, NY, USA) with AxioVision software. Liver steatosis was assessed according to the percentage of the macro- and micro-vesicular fat vacuoles at 20 X magnification in four fields, using Image J software (NIH, Bethesda, MD, USA), as was described previously (40). Briefly, the original images were converted to 8-bit black-and-white, followed by black and white inversion. Then, an upper threshold of the grayscale was applied to the converted images. Subsequently, a particle analysis was conducted by using the “Analyze Particles” function of Image J. A circularity parameter of 0.5–10 was set to remove any potential noise that are not lipid droplets.

### RNA isolation, cDNA synthesis, and quantitative polymerase chain reaction

Liver mRNA extraction was performed using the PureLink RNA Mini Kit (Thermo Fisher Scientific) as reported previously (13, 41). Weighted liver tissues (approximately 100 mg) were homogenized in a lysis buffer with 1% 2-mercaptoethanol and centrifuged at 2,600 x *g* for 5 min. Then, the supernatant was transferred to an RNase-free tube and washed with 70% ethanol. The entire mixture was filtered by the spin cartridge and washed with two different wash buffers. A total of 100 μL RNase-free water was added to the center of the spin cartridge at three separate times; the mixture was incubated at room temperature for 1 min, followed by centrifugation at 12,000 x *g* for 2 min to elute the liver mRNA. Liver cDNA synthesis was performed by using Novo cDNA Kit (BioVision, Minneapolis, CA, USA) according to the manufacturer’s instructions. Each reaction contained 500 ng mRNA samples, 1 μL random primer, 1 μL dNTP, 5 μL RT buffer, 0.5 μL RNase inhibitor, and 1 μL RTase in a Biometra TAdvanced 96G Thermal Cycler (Analytik Jena, Jena, Germany). The program conditions were 25°C for 10 min, 42°C for 50 min, and 85°C for 5 min. The quantification of liver cDNA was carried out by mixing the 10-fold diluted cDNA with 10 μL 2X PowerUp SYBR Green Master Mix, 2 μL of 10 μM primer mix that includes forward and reverse primers, and 3 μL PCR water. The cycling conditions were described previously (13). Primer sequences were listed in Supplementary Table 2.

### Protein extraction and western blot

Protein was extracted from whole cell lysates of liver tissue (approximately 50 mg) by homogenizing the tissues in a radioimmunoprecipitation assay buffer (Thermo Fisher Scientific) containing 1% protease inhibitors. Subsequently, the homogenates were centrifuged at 25,200 x *g* for 30 min at 4°C, and the supernatants were collected for further analyses. Liver nuclear protein was extracted by using the Nuclear Extraction Kit (Abcam). Briefly, 100 mg frozen liver tissues were homogenized in a pre-extraction buffer and incubated on ice for 15 min. After centrifugation for 10 min at 16,128 x *g* at 4°C, an extraction buffer containing 1% dithiothreitol (DTT) and a protease inhibitor cocktail was added. The entire mixture was placed on ice for 15 min, followed by centrifugation at 4°C for 10 min. The concentration of the proteins was quantified by using the Pierce Rapid Gold BCA Protein Assay Kit (Thermo Fisher Scientific).

The detailed western blot protocol was described in our previous publications (13, 41). The primary antibodies including acetyl coenzyme A carboxylase alpha (ACCα), diacylglycerol *O*-acyltransferase 2 (DGAT2), fatty acid synthase (FAS), stearoyl-CoA desaturase-1 (SCD-1), acyl-CoA oxidase 1 (ACOX1), carnitine palmitoyltransferase-II (CPT-II), sterol regulatory element-binding protein 1 (SREBP-1), peroxisome proliferator-activated receptor gamma coactivator 1-alpha (PGC-1α), and AMP-activated protein kinase (AMPK) were mouse obtained from Santa Cruz Biotechnology (Dallas, TX, USA); phospho-AMPK (Thr172, p-AMPK), and peroxisome proliferator-activated receptor alpha (PPARα) were obtained from ABclonal (Woburn, MA, USA). The antibodies from Santa Cruz Biotechnology were 1:1000 diluted with 5% bovine serum albumin (BSA, Thermo Fisher Scientific). The antibodies from ABclonal were 1:2000 diluted with 3% non-fat milk (Research Products International, Mt Prospect, IL, USA). An anti-mouse secondary antibody (1:1000 5% BSA) or anti-rabbit secondary antibody (1:500 3% non-fat milk) was applied to the membranes that were blotted with primary antibodies from Santa Cruz Biotechnology or ABclonal, respectively.

Briefly, 50 μg protein was loaded to the gel. We utilized 4–12% Bis-Tris gels with MOPS running buffer for proteins with a low to medium molecular weight (DGAT2, SCD-1. ACOX-1, CPT-II, p-AMPK, AMPK, and PPARα), while 3–8% tris-acetate gels with tris-acetate SDS Running Buffer were utilized to separate high molecular weight proteins (MTP, FAS, ACCα, SREBP-1, and PGC-1α). Gels and running buffers were purchased from Thermo Fisher Scientific. Electrophoresis was conducted per manufacturer’s instructions. A dry transfer system (iBlot2) was employed to transfer proteins from gels to nitrocellulose membranes. This was followed by non-specific blocking with 5% bovine serum albumin (BSA) for 1 h. After three washes, the membranes were incubated in the primary antibodies at 4°C overnight. If the primary antibodies were not conjugated with horseradish peroxidase (HRP), a secondary antibody was applied. Membranes were developed for visualization with the addition of chemiluminescent reagents and the signals were extracted with a UVP ChemStudio Imaging System (Analytik Jena). The signal intensity of all blots was analyzed by using Image J software. β-Actin was used as a loading control. The signal intensity ratios between the target proteins and β-actin were calculated and further analyzed.

### Extraction of carotenoids and high-performance liquid chromatography

β-Apo-8′-carotenal (purity ≥ 96%, catalog number: 10810, Sigma-Aldrich, St. Louis, MO, USA) was dissolved in HPLC-grade acetone and added to the samples as an internal control before carotenoid extraction. A total of 80 mg dietary pellets were placed in the ZR Bashing Bead Lysis Tubes (Zymo Research, Irvine, CA, USA). HPLC-grade acetone (500 μL) was added to each tube, followed by a vibrant vortex and centrifugation for 5 min at 16,000 x *g* at 4°C. Individual liver tissue (approximately 60 mg) was homogenized in 1 mL HPLC-grade acetone and centrifuged at 16,000 x *g* at 4°C for 10 min. For serum carotenoid extraction, 60 μL pooled serum from the co-housed mice was extracted with a mixture of petroleum ether (PE), acetone, and butylated hydroxytoluene (BHT) (PE: acetone = 3:1:1% BHT), followed by centrifugation at 1,792 x *g* for 1 min. All the supernatants were collected and dried under nitrogen gas. Carotenoids in the dietary pellets were reconstituted in 80 μL acetone, whereas liver carotenoids and serum carotenoids were reconstituted in 200 and 60 μL acetone, respectively.

The reconstituted carotenoids (5 μL) were detected by an Ultimate 3000 HPLC (Thermo Fisher Scientific). Varying concentrations of pure α-carotene (purity ≥ 97%, catalog number: 50887, Sigma-Aldrich) and β-carotene (purity ≥ 97%, catalog number: 217538, Millipore Sigma, Burlington, MA, USA) were used as external controls. The channels used for β-apo-8′-carotenal was 459 nm, for β-carotene was 455 nm, and for α-carotene was 449 nm. The HPLC conditions were described previously (42). Briefly, the column was Acclaim C30, 5 μm, 4.6 × 150 mm. The gradient of the mobile phase was set as follows: acetonitrile: methanol: methyl tert-butyl ether (v/v/v: 25:75:0 at time 0–20 min, 15:35:50 at time 20–25.5 min, 25:75:0 at time 25.5–30 min). The flow rate for the mobile phase was 1.0 mL/min.

### PPARα transcription factor activity assay

The activity of PPARα was measured using a commercialized kit (Abcam) according to the manufacturer’s protocol. Following nuclear protein extraction and protein concentration quantification, 90 μL Assay Buffer was added to a 96-well plate containing a specific double-stranded DNA probe with the peroxisome proliferator response element (PPRE) immobilized at the bottom. Subsequently, 10 μL liver nuclear proteins were added. The plate was sealed and incubated overnight at 4°C without agitation. Then, a diluted PPARα primary antibody (1:100 with Assay Buffer) was added to the plate, followed by incubation for 1 h at room temperature. After washing the plate with the supplied wash buffer, we added a secondary antibody, followed by an anti-rabbit HRP conjugate. Following an hour of incubation, 100 μL Stop Solution was added to end the reaction, and the plate was read at OD 450 nm.

### Statistical analysis

Normality of distribution of the data was assessed using the D’Agostino-Pearson omnibus normality test with *p* > 0.05 considered normally distributed. Equality of data variance was examined by using an *F* test with *p* > 0.05 considered equal variances. One-way ANOVA with *post hoc* Tukey HSD was utilized to compare the differences between multiple groups. Kruskal-Wallis test with *post hoc* Tukey HSD was used to compare the differences of non-parametric parameters between multiple groups. Two-way mixed ANOVA with *post hoc* Tukey HSD was employed to depict the difference between multiple groups by including time as a factor. Statistical significance was set as *p* < 0.05. All statistical analysis was performed using GraphPad Prism 9 (San Diego, CA, USA). Values are listed in the text and figures as mean ± standard error of the mean (SEM).

## Results

### Carotenoid composition-diet pellets, serum, and liver

Carrots are the main dietary sources of α-carotene and β-carotene (43); thus, we measured the concentration of these carotenoids using HPLC as described in the methods section. LFD and HFD pellets did not contain either α-carotene or β-carotene (Table 1A). The incorporation of the white carrots essentially acted as a carrot carotenoid control group as they were integrated into the dietary pellets at the same percentage. Therefore, the components of the white carrot and orange carrot pellets are similar, except for these carotenoids. Consistently, the HFD + WC pellet consisting of 20% white carrot powder contained no detectable α-carotene and a negligible amount of β-carotene (0.002 μmol/g). The HFD + OC pellet consisting of 20% orange carrot powder was determined to contain a similar amount of α-carotene and β-carotene at concentrations of 0.12 and 0.11 μmol/g, respectively.

**TABLE 1:** α-Carotene and β-carotene concentrations in panel (A) diet pellets (*n* = 3), (B) serum (LFD: *n* = 11; HFD, HFD + WC, HFD + OC: *n* = 12), and (C) liver (LFD: *n* = 11; HFD, HFD + WC, HFD + OC: *n* = 12).

Treatment groups	(A) Diet pellets (μmol/g)		(B) Serum (μ M)		(C) Liver (μ mol/g)	
			
	α -Carotene	β -Carotene	α -Carotene	β-Carotene	α-Carotene	β-Carotene
LFD	0.0	0.0	0.0	0.0	0.0	0.0
HFD	0.0	0.0	0.0	0.0	0.0	0.0
HFD + WC	ND	0.002	ND	ND	ND	ND
HFD + OC	0.12 ± 0.01	0.11 ± 0.01	0.52 ± 0.20	1.43 ± 0.10	3.30 ± 1.90	8.04 ± 3.00

ND, not detected. Values are means ± SD. Diet pellets were administered ad libitum.

In the circulation, α-carotene and β-carotene were not detected in the LFD and HFD groups since these compounds were not present in the diets (Table 1B). Notably, neither α-carotene nor β-carotene was detected in the serum of the HFD + WC fed group. Both α-carotene and β-carotene were detected in the serum of the HFD + OC fed group with a higher concentration of β-carotene (1.4 ± 0.1 μM) compared to the concentration of α-carotene (0.5 ± 0.2 μM). We measured the hepatic concentration of α-carotene and β-carotene since liver is the main storage of carotenoids including α-carotene, β-carotene, lycopene, lutein, zeaxanthin, and β-cryptoxanthin (44, 45). We observed neither α-carotene nor β-carotene in the LFD, HFD, and HFD + WC groups (Table 1C), which was in line with the minimal carotenoid content in the diet pellet and circulation. α-Carotene and β-carotene were measured in HFD + OC group at concentrations of 3.3 ± 1.9 and 8.0 ± 3.0 μmol/g, respectively, in line with earlier reports that β-carotene was found in higher concentrations than α-carotene in liver (44, 45). The chromatograms of α-carotene and β-carotene within the diet pellet, serum, and liver samples are provided in Supplementary Figure 1.

### Carotenoids in orange carrot significantly reduced body weight

**FIGURE 1:**
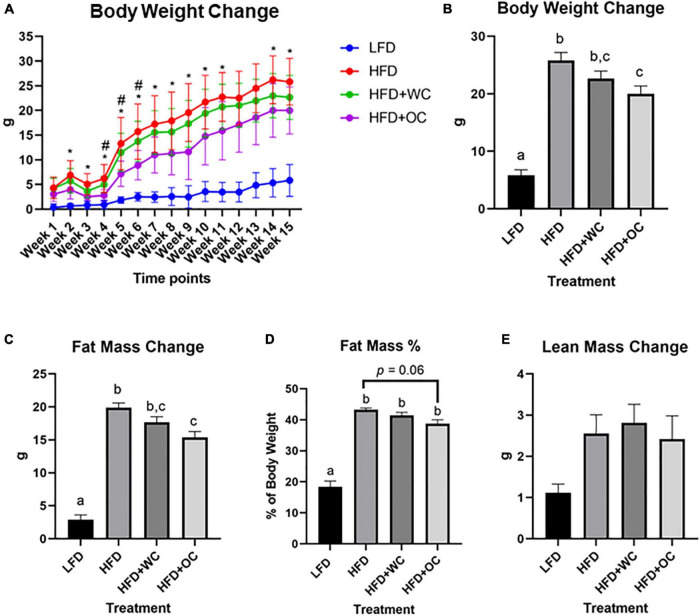
Orange carrot-rich diet inhibited HFD-induced changes in body composition. LFD: *n* = 11; HFD, HFD + WC, HFD + OC: *n* = 12. **(A)** Weekly changes in body weight compared to baseline (week 0), *HFD vs. HFD + OC, *p* < 0.05; #HFD + WC vs. HFD + OC, *p* < 0.05. **(B)** Body weight change at endpoint (week 15) compared to baseline. **(C)** Change in fat mass determined by EchoMRI. **(D)** Fat mass percentage of Week 15 body weight. **(E)** Change in lean mass determined by EchoMRI. Values are means ± SEM. Body weight change was analyzed by two-way mixed ANOVA with *post hoc* Tukey HSD. Changes of body weight, fat mass and lean mass were analyzed by one-way ANOVA with *post hoc* Tukey HSD. Letter difference indicate statistical significance (*p* < 0.05).

We monitored the weight gain throughout the study. The body weights of the HFD + OC became significantly lower than the HFD group from Week 2, which persisted for the duration of the study (*p* < 0.05), except for Weeks 12 and 13 (Figure 1A). There was a continuous trend of lower body weight changes in the HFD + OC group compared to HFD + WC that reached statistical significance at Weeks 4, 5, and 6 (*p* < 0.05). Food consumption is reported in Supplementary Figure 2. Following Week 15 and prior to necropsy, body weight was recorded a final time (Figure 1B). On average, mice in the HFD gained significantly more body weight than the LFD group (25.8 ± 3.4 g vs. 5.8 ± 2.5 g, *p* < 0.01); HFD + WC was not significantly different from HFD. HFD + OC was significantly lower than HFD (20.0 ± 4.0 g vs. 25.8 ± 3.4 g, *p* < 0.05) and was trending to be lower than HFD + WC, but this difference was not statistically significant.

Consistent with the pattern of body weight gain, the Echo-MRI results depicted a significantly higher fat mass increase in the HFD than in the LFD group (19.9 ± 0.7 g vs. 2.9 ± 0.7 g, *p* < 0.01). At the same time, the HFD + WC was not significantly different from the HFD group. HFD + OC was significantly lower than HFD (15.4 ± 0.9 g vs. 19.9 ± 0.7 g, *p* < 0.01) and was trending to be lower than HFD + WC, but this difference did not reach statistical significance (Figure 1C). The fat mass percentage of total body weight was measured, of which the HFD, HFD + WC, and HFD + OC groups were significantly higher than the LFD group (Figure 1D). The decrease in fat mass percentage from HFD conditions seen in the HFD + OC group nearly reached significance (*p* = 0.06). The changes in the lean mass amongst the groups were not statistically significant (Figure 1E).

### Carotenoids in orange carrots inhibited NAFLD severity

#### Hepatic steatosis

**FIGURE 2:**
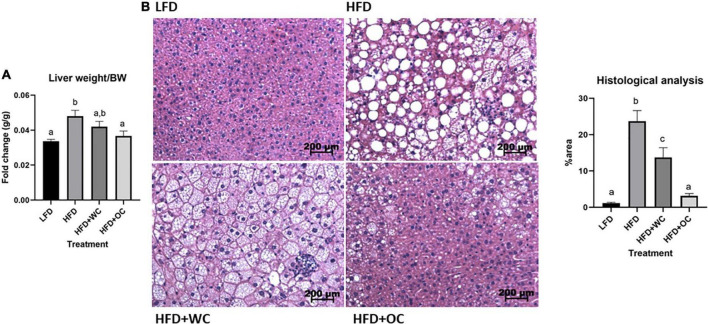
Orange carrot-rich diet improved status of HFD-induced hepatic steatosis. **(A)** Graphical representation of liver weight-to-body weight (BW) ratio. LFD: *n* = 11; HFD, HFD + WC, HFD + OC: *n* = 12. **(B)** Hematoxylin and eosin (H&E) staining of livers, percentage of area covered by lipid deposits at 20 X magnification. Liver weight was analyzed by one-way ANOVA with *post hoc* Tukey HSD. Liver histology was analyzed by using the Kruskal-Wallis test with *post hoc* Tukey HSD. Letter differences indicate statistical significance (*p* < 0.05).

Liver weights depicted a similar trend to the overall body weight (Figure 2A). The HFD treatment group had a significantly higher liver weight-to-body weight (LW/BW) ratio than the LFD group. The LW/BW ratio of the HFD + WC group decreased from the HFD group but was not significantly different; additionally, this decrease reached a level statistically comparable to the LFD group. Finally, the LW/BW ratio of the HFD + OC group was significantly lower than the ratio of the HFD group, though not significantly different from the HFD + WC ratio. Notably, orange carrot supplementation sufficiently decreased the liver weight/body weight ratio in the HFD + OC group to a point comparable to LFD, like the HFD + WC group but to a greater degree.

H&E staining depicted the extent of fat deposits within the livers (Figure 2B). The LFD liver histology showed a very minimal presence of fat. The HFD livers were riddled with fatty deposits that were large in both size and number, which covered a significantly larger percentage of area than the LFD livers (23.7 ± 2.9% vs. 1.2 ± 0.2%, *p* < 0.01). The HFD + WC livers still contained fat throughout the tissues (13.8 ± 2.6%), but not to the extent of the HFD livers (*p* < 0.01). However, in the HFD + OC group, the area of livers covered by lipid droplets (3.2 ± 0.6%) was significantly reduced compared to both HFD and HFD + WC (*p* < 0.01) and to a level comparable to LFD.

#### Triglycerides and free fatty acids in the liver and the circulation

**FIGURE 3:**
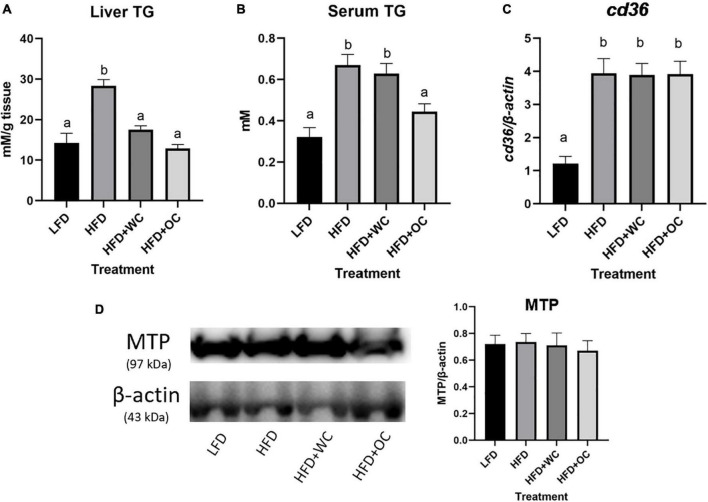
Hepatic and circulatory fat content. **(A,B)** Triglyceride (TG) content in panel **(A)** liver and **(B)** serum. For both liver and serum TG assay, LFD, HFD, HFD + WC, HFD + OC: *n* = 10. **(C)** Changes in mRNA levels in hepatic *cd36* generated from qPCR. **(D)** Western blot of hepatic MTP protein expression performed with 3–8% tris-acetate gel, graphical changes between the dietary groups. For both western blot and qPCR assays, LFD: *n* = 11; HFD, HFD + WC, HFD + OC: *n* = 12. Values are means ± SEM. Data were analyzed by one-way ANOVA with *post hoc* Tukey HSD. Letter differences indicate statistical significance (*p* < 0.05).

Based on the differential presence of fatty deposits within the livers throughout the treatment groups, hepatic triglyceride (TG) levels were measured (Figure 3A). The HFD livers contained a significantly higher content of TG than the LFD livers (28.3 ± 1.5 mM/g tissue vs. 14.3 ± 2.4 mM/g tissue, *p* < 0.01), and a significant reduction in TG content was observed in the HFD + WC livers (17.6 ± 0.9 mM/g tissue) compared to the HFD group (*p* < 0.01), which was further reduced in the HFD + OC livers (12.9 ± 0.1 mM/g tissue, *p* < 0.01). However, the difference in TG content between the HFD + WC and HFD + OC groups was not significant. Circulatory TG levels were investigated by measuring triglyceride content within the serum (Figure 3B). As expected, TG content in the HFD group was significantly higher than in the LFD group (0.7 ± 0.1 mM vs. 0.3 ± 0.1 mM, *p* < 0.01). Serum TG in the HFD + WC group (0.6 ± 0.1 mM) was significantly higher than LFD (*p* < 0.01) and comparable to the HFD group. Notably, the serum TG content was significantly reduced in the HFD + OC (0.4 ± 0.04 mM) compared to both HFD and HFD + WC groups (*p* < 0.01, *p* = 0.04, respectively), and such decrease was brought down to a level comparable to the LFD group.

To investigate how serum TG was associated with the TG content in the liver, fatty acid transport cluster of differentiation 36 (CD36) and microsomal triglyceride transfer protein (MTP) were examined. As a result, the LFD livers contained the least mRNA amount of *cd36* compared to HFD, HFD + WC, and HFD + OC (*p* < 0.01) (Figure 3C). This trend of higher *cd36* content is expected in these HFD due to the increased composition (60%) of fat within the diet compared to LFD (10%). The protein expression of MTP within the liver was quite consistent across the groups with no significant differences (Figure 3D). These data indicate that the higher lipid accumulation in the HFD liver might be due to the increased hepatic fatty acid intake, not disturbed fatty acid output.

#### Fatty acid synthesis

**FIGURE 4:**
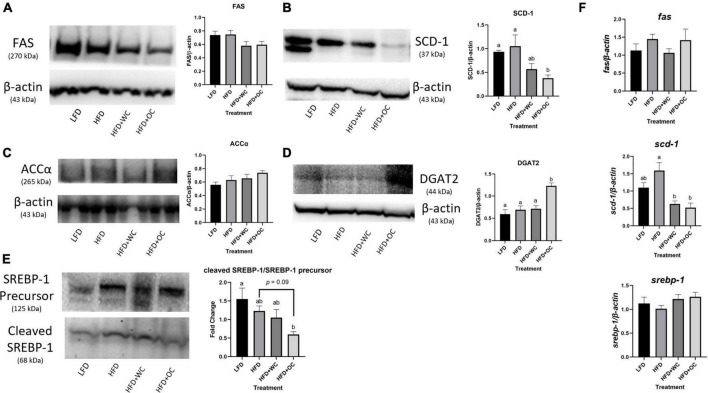
Carotenoids minimally inhibit hepatic fatty acid synthesis. Western blot of hepatic **(A)** FAS, **(B)** SCD-1, **(C)** ACCα, **(D)** DGAT2, and **(E)** ratio of cleaved SREBP-1 to SREBP-1 precursor protein expression; blots in panels **(A,C,E)** performed with 3–8% tris-acetate gels; blots in panels **(B,D)** performed with 4–12% Bis-Tris gels; graphical changes between the dietary groups. **(F)** Changes in mRNA levels in hepatic *fas*, *scd-1*, and *srebp-1* generated from qPCR. For both western blot and qPCR, LFD: *n* = 11; HFD, HFD + WC, HFD + OC: *n* = 12. The fold change of mRNA and protein levels were analyzed by one-way ANOVA with *post hoc* Tukey HSD. Letter differences indicate statistical significance (*p* < 0.05).

In order to look into the changes observed in the histology and triglyceride experiments, genes and proteins related to fatty acid synthesis were investigated. Fatty acid synthase (FAS) is a major player in this process as the enzyme catalyzes the *de novo* synthesis of fatty acids (46). Protein expression of FAS in the LFD and HFD were comparable, which were slightly higher than the carrot groups. However, there were no significant differences between all the treatment groups (Figure 4A). Additionally, this study did not lead to significant changes in *fas* at the mRNA level (Figure 4F).

Stearoyl-CoA desaturase-1 (SCD-1) is involved in fatty acid synthesis by catalyzing the generation of monounsaturated fatty acids (MUFAs), such as oleate and palmitoleate formed *via* desaturation of stearoyl-CoA and palmitoyl-CoA, respectively (47). Firstly, the LFD and HFD groups did not differ significantly (Figure 4B). HFD + WC led to a drop in SCD-1 expression, but this was non-significant compared to both LFD and HFD groups. Notably, the HFD + OC significantly decreased SCD-1 compared to the HFD (*p* < 0.01), which was not achieved by the HFD + WC. While the difference between the carrot-fed groups was not significant, a lower SCD-1 expression can be observed in the HFD + OC. The mRNA levels of SCD-1 depicted similar trends as the protein expression. Analysis achieved *via* qPCR portrayed that mRNA levels of *scd-1* were differentially expressed across the treatment groups (*p* < 0.01), and the pattern was consistent with the protein expression (Figure 4F).

Protein expression of acetyl coenzyme A carboxylase alpha (ACCα) was also investigated due to its role in catalyzing the carboxylation of acetyl-CoA to form malonyl-CoA (48). ACCα did not appear to be highly expressed in the liver, and no significant trends were observed amongst the treatment group (Figure 4C). Finally, protein expression of diacylglycerol O-acyltransferase 2 (DGAT2) was investigated due to its role in synthesizing triglycerides by covalently binding diacylglycerol to long-chain fatty acyl-CoAs (49). Most of the groups did not have differential expression of DGAT2 as the changes between LFD, HFD, and HFD + WC were not significant (Figure 4D). Surprisingly, the HFD + OC group expressed a significantly higher content of DGAT2 compared to all the other dietary groups (*p* < 0.01).

Sterol regulatory-element binding proteins (SREBPs) are a family of transcription factors responsible for regulating lipid biosynthesis, and adipogenesis *via* enzymes involved in cholesterol, fatty acid, triacylglycerol, and phospholipid synthesis (50). The activity of SREBP-1 is dependent on the cleavage of its precursor compound; thus, the ratio of cleaved SREBP-1/SREBP-1 portrays the relative activity within that group. This ratio was comparable in the LFD, HFD, and HFD + WC groups (Figure 4E). The HFD + OC group had the lowest ratio (HFD + OC vs. LFD, *p* < 0.01; HFD + OC vs. HFD, *p* = 0.09), indicating the least amount of SREBP-1 activity. At the same time, this change did not reach a significant decrease compared to the HFD and HFD + WC groups; this ratio was significantly lower than the LFD group. There were no observed significant differences in *srebp-1* between the groups at the mRNA level (Figure 4F).

#### β-Oxidation

**FIGURE 5:**
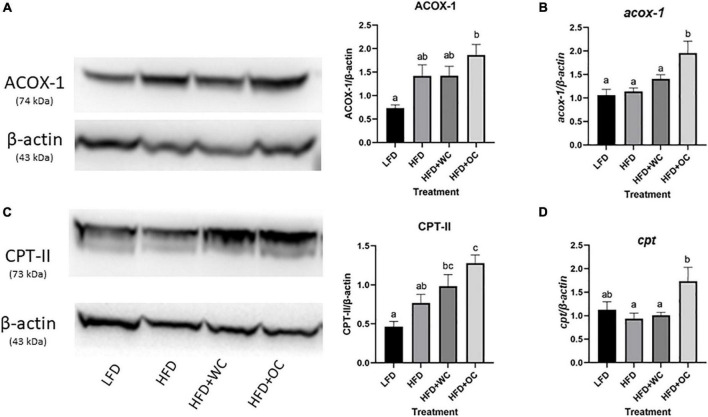
Carotenoids in HFD + OC diet promote hepatic β-oxidation. Western blot of **(A)** ACOX-1 and **(C)** CPT-II protein expression; performed with 4–12% Bis-Tris gels; graphical changes between the dietary groups. Changes in mRNA levels in hepatic **(B)** *acox-1* and **(D)** *cpt* generated *via* qPCR. For both western blot and qPCR, LFD: *n* = 11; HFD, HFD + WC, HFD + OC: *n* = 12. The fold change of mRNA and protein levels were analyzed by one-way ANOVA with *post hoc* Tukey HSD. Letter differences indicate statistical significance (*p* < 0.05).

To further investigate hepatic lipid regulation, we explored β-oxidation related targets, including acyl-CoA oxidase 1 (ACOX1) and carnitine palmitoyltransferase-2 (CPT-II) within the fatty acid β-oxidation pathway. The HFD + OC group had the most robust ACOX1 protein expression, significantly higher than the LFD group (*p* < 0.01) (Figure 5A). Despite the non-significant difference, there was a pattern of higher ACOX1 in the HFD + OC group compared to other groups. The protein content of ACOX1 portrayed a slightly different story than the mRNA levels. Most of these treatment groups did not exhibit differential expression of *acox1* mRNA levels as the changes between LFD, HFD, and HFD + WC were not significant (Figure 5B). Notably, the HFD + OC group expressed a significantly higher content of *acox1* compared to all the other treatment groups (*p* < 0.01).

The LFD and HFD livers expressed comparable levels of CPT-II protein content. HFD + WC had a slightly higher but insignificant increase in expression than HFD (Figure 5C). HFD + OC livers had the strongest expression of CPT-II, significantly higher than both LFD and HFD groups (*p* < 0.01, *p* = 0.0149, respectively). Despite the lack of significance, the HFD + OC CPT-II expression trended higher than the HFD + WC group. The *cpt* mRNA content was consistent throughout most groups as the LFD, HFD, HFD + WC levels were comparable (Figure 5D). Notably, there was a significant increase in *cpt* within the HFD + OC group compared to the HFD and HFD + WC groups (*p* = 0.02, *p* < 0.01, respectively).

### Potential underlying pathways

**FIGURE 6:**
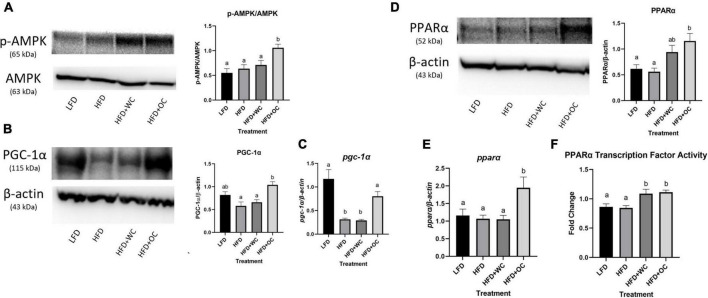
Carotenoids in HFD + OC diet enhance regulators of hepatic lipid metabolism. **(A)** Western blot of phosphorylated AMPK (p-AMPK) and total AMPK protein expression, performed with 4–12% Bis-Tris gel; statistical analysis of p-AMPK/AMPK ratio. **(B)** Western blot of PGC-1α protein expression, performed with 3–8% tris-acetate gel. **(C)** Changes in mRNA levels in hepatic *pgc-1*α. **(D)** Western blot of PPARα protein expression, performed with 4–12% Bis-Tris gel. **(E)** Changes in mRNA levels in hepatic *ppar*α. For both western blot and qPCR, LFD: *n* = 11; HFD, HFD + WC, HFD + OC: *n* = 12. **(F)** Graphical changes in nuclear PPARα transcription factor activity. LFD, HFD, HFD + WC, HFD + OC: *n* = 10. Values are means ± SEM. The fold change of mRNA, protein, and PPARα transcription factor activity were analyzed by one-way ANOVA with *post hoc* Tukey HSD. Letter differences indicate statistical significance (*p* < 0.05).

As phosphorylation of AMPK is required for activation of downstream regulation (51–53), protein levels of the p-AMPK/total AMPK ratio within the liver were assessed (Figure 6A). The LFD and HFD groups expressed comparable levels of the p-AMPK/AMPK ratio. The HFD + WC group exhibited a slight increase in p-AMPK/AMPK compared to the LFD and HFD groups, but this difference was not significant in measure. The HFD + OC group had a further increase in the p-AMPK/AMPK ratio that was significantly higher than the LFD, HFD, and HFD + WC groups (*p* < 0.01, *p* < 0.01, *p* = 0.03, respectively).

AMPK activity can promote the activity of PPARs by upregulating peroxisome proliferator-activated receptor gamma coactivator 1-alpha (PGC-1α) (54). The PGC-1α protein expression was consistent throughout most groups as the LFD, HFD, HFD + WC levels were comparable (Figure 6B). Notably, there was a significant increase in PGC-1α within the HFD + OC group compared to the HFD and HFD + WC groups (*p* < 0.01). The mRNA levels depicted similar trends to protein expression as *pgc-1*α in the HFD and HFD + WC groups were significantly decreased from LFD (*p* < 0.01) (Figure 6C). The HFD + OC group restored the *pgc-1*α to a statistically comparable level with the LFD group.

Consistently, the protein content of PPARα within the LFD and HFD groups were similar (Figure 6D). The HFD + WC group exhibited a slightly greater expression of PPARα than the LFD and HFD groups, but this difference was not significant in measure. Finally, PPARα expression was the strongest in the HFD + OC group; this change was significant compared to the LFD and HFD groups (*p* < 0.01), but not against the HFD + WC group. At the mRNA level, *ppar*α was similar across the LFD, HFD, HFD + WC groups but significantly higher in the HFD + OC group (*p* < 0.05) (Figure 6E). To further determine the influence of PPARα within the liver, PPARα transcription factor activity was investigated within nuclear protein extracts (Figure 6F). PPARα transcription factor activity within the HFD + OC was comparable with the HFD + WC group, but statistically higher than those within the LFD and HFD groups (*p* = 0.04, *p* = 0.03, respectively).

## Discussion

Throughout the study, the orange carrot diet displayed more potent efficacy in reducing the HFD-induced body weight gain than the white carrot diet. In a clinical study by Takagi et al. (16), subjects were randomly assigned to one of the four diets: high lycopene + high lutein, high lycopene + low lutein, low lycopene + high lutein, and low lycopene + low lutein. At the end of the study, the visceral fat level was significantly decreased in all the dietary groups. However, a significant decrease in waist circumference was only observed in the high lycopene + high lutein (16). Such data were consistent with the results of our study, showing that the dietary carotenoids in the vegetables may add another layer of protective effect against lipogenesis. However, the study by Takagi et al. employed lycopene and lutein. To our best knowledge, till date, no study has used the same methodology to explore the effects of alpha- and beta-carotene, in whole food.

α-Carotene and β-carotene are among the most frequently consumed dietary carotenoids in North American diets (55). Liver is the major storage organ for dietary carotenoids, including α-carotene, β-carotene, lycopene, β-cryptoxanthin, lutein, and zeaxanthin, while β-carotene is one of the most abundant carotenoids, other being lycopene (44, 45, 56). In this study, the average serum and hepatic β-carotene concentrations of the orange carrot supplemented mice were 1.4 ± 0.1 μM and 8.0 ± 3.0 μmol/g tissue, respectively. The average hepatic α-carotene concentration of the orange carrot supplemented mice was 3.3 ± 1.9 μmol/g tissue, and the serum α-carotene concentration was not detected due to the extremely low circulating α-carotene concentration that was expected at a nanomolar level (57). These concentrations fall within the range of typical β-carotene concentrations in human serum (0.04–2.26 μM) and livers (0.39–19.4 μmol/g tissue), and human hepatic α-carotene concentration (0.075–10.8 μmol/g) (55). Oral β-carotene supplementation or the consumption of a high-β-carotene diet may lead to a higher circulating β-carotene concentration at 0.68–2.26 μM (58, 59). We recently engineered *S. boulardii* that could synthesize high doses of β-carotene, so the intake of such engineered probiotics may lead to an even higher β-carotene concentration in the circulation (42), indicating that the dosage of supplemented dietary carotenoids through carrots was of physiological relevance.

As provitamin A carotenoids, α-carotene and β-carotene can be enzymatically cleaved at the 15-, 15′-double bond by β-carotene dioxygenase 1 (BCO1) to produce all-trans retinal (vitamin A aldehyde) (9). In the liver, all-trans retinal can be either reversibly reduced to all-trans retinol and then esterified to retinyl esters for storage, or irreversibly oxidized to all-trans retinoic acid (ATRA), the biologically active form of vitamin A (60). It has been reported that the hepatic ATRA and retinol concentrations in mice livers were at the pmol/g tissue range (61), which was beyond the limit of detection of our carotenoid quantification method. Therefore, it is unknown whether orange carrot supplementation results in higher levels of hepatic vitamin A and ATRA. As the biologically active form of vitamin A, ATRA is a high-affinity ligand for retinoic acid receptors, while its isomer, 9-*cis*-retinoic acid (9cRA) is an agonist of retinoid X receptors (62–64). However, we were unable to detect 9cRA in livers and serum of the animals. Since we did not observe a significant change in hepatic RXRα and RARβ expressions (Supplementary Figure 3), and the hepatic RXRβ concentration was undetectable (data not shown), the beneficial effects of orange carrots may stem from α- and β-carotene as parent compounds, not ATRA.

Hepatic steatosis may result from excessive fatty acid or TG uptake, reduced TG output, increased *de novo* lipogenesis, decreased β-oxidation, or a combination. In the HFD group, we observed promoted levels of serum TG, which was intriguingly significantly correlated with higher liver TG levels (*p* < 0.01) (Supplementary Figure 4). CD36 mediates long-chain fatty acid uptake in liver (65). In this study, the expressions of *cd36* mRNA were higher in mice fed on the HFD diet, despite white carrot or orange carrot supplementation, indicating that the increased hepatic uptake of FFA from the circulating system played a vital role in developing HFD-induced liver steatosis. MTP is rate-limiting for the assembly of apoB-containing lipoprotein and hepatic TG secretion (66, 67), so the comparable MTP protein expressions across the dietary groups suggested that hepatic TG output minimally contributed to the development of NAFLD.

The orange carrot supplementation was more efficient in alleviating HFD-induced NAFLD than the supplementation of white carrots. Such result is in line with a previous publication that the consumption of spinach and tomato (both containing high levels of carotenoids) efficiently ameliorated NAFLD in rats fed with the HFD (68). However, the positive control of that study was a lower percentage of carotenoid-rich vegetables, leading to a proportionally lower concentration of other beneficial compounds, such as fiber. Therefore, it is unknown whether the anti-NAFLD efficacy of that diet was mainly attributed to carotenoids or fiber. The advantage of the current study is that we employed white carrot as a positive control, which contains an extremely low level of carotenoids, but an equivalent amount of fiber, compared to the orange carrot. Thus, we are confident that the NAFLD-preventive efficacy of the orange carrot diet was primarily from α-carotene and β-carotene, not fiber. ACCα is the rate-limiting enzyme in regulating fatty acid synthesis (48, 69). In the current study, the ACCα protein levels did not differ among the groups. Although we observed a pattern of decreased FAS and significantly reduced SCD-1 in the HFD + OC group, DGAT-2 significantly increased in the HFD + OC group. Therefore, it is inconclusive to address whether orange carrot supplementation ameliorated NAFLD through modulating fatty acid synthesis. ACOX1 catalyzes the first and rate-determining step in peroxisomal fatty acid oxidation (70), and the mutation of ACOX1 was shown to induce NAFLD progression and exacerbate hepatocellular damage (71). In the current study, ACOX1 was significantly improved in the mice with orange carrot supplementation, which was in line with the report that the consumption of foods high in β-carotene and other carotenoids increased hepatic ACOX1 in rats (68). CPT-II is one of the key enzymes in the mitochondrial β-oxidation of fatty acids (72). A significantly enhanced CPT-II protein in the HFD + OC group indicates that promoted fatty acid β-oxidation may be the primary mechanism of an orange carrot-rich diet that prevented HFD-induced NAFLD development.

By further exploring the molecular targets involved in β-oxidation, we found a significantly increased p-AMPK expression in the HFD + OC group compared with the HFD group. AMPK is a heterotrimeric complex, and its α subunit is the main catalytic domain (53). Although the α subunit can be phosphorylated at Thr172, Thr258, and Ser485 sites, phosphorylation of Th172 is the hallmark of AMPK activation (51–53). Previous studies have shown that the activation of AMPK could protect against diet-induced NAFLD and NASH (33, 73). AMPK phosphorylation can inhibit the cleavage and maturation of SREBP-1, subsequently attenuating hepatic steatosis through regulating lipogenic genes (74), so it is possible that the dietary carotenoids in the orange carrot could reduce SRBEP-1 cleavage through promoting the phosphorylation of AMPK. In addition to AMPK activation, we observed significantly higher hepatic levels of *ppar*α mRNA, PPARα protein, and PPARα transcription factor activity in the mice supplemented with orange carrots, indicating that the dietary carotenoids in orange carrots might prevent the development of NAFLD by targeting the PPARα pathway. PPARα, PPARβ/δ, and PPARγ are three PPAR isoforms ubiquitously expressed in various tissues, but PPARα is mainly present in liver (75). In NAFLD patients, the hepatic PPARα expression was negatively correlated with occurrence of NASH, severity of NAFLD, ballooning of the hepatocytes, and NASH activity score and fibrosis (76). Ip et al. found that in mice, PPARα knockout resulted in significantly more severe steatohepatitis, while the administration of Wy-14643, a potent PPARα agonist, substantially prevented diet-induced NAFLD and liver injury (77). Furthermore, the injection of Wy-14643 promoted expression of acyl-CoA oxidase (77), which was in line with our finding that the increased PPARα was associated with an elevated level of ACOX1 in the HFD + OC group. PGC-1α acts as a coactivator of PPARα and promotes PPARα-mediated transcriptional activity in modulating its target genes, such as genes involved in β-oxidation (78). Interestingly, in this study, we found a more potent increase in PGC-1α protein expression in the HFD + OC group, compared with the HFD + WC group, indicating that the dietary carotenoids in the orange carrots may activate the PGC-1α-PPARα pathway. In summary, our study reveals that the dietary carotenoids in the orange carrots rich in dietary carotenoids, specifically α-carotene and β-carotene, may regulate the fatty acid synthesis and β-oxidation-related genes by activating AMPK and PPARα. However, how these compounds promote the phosphorylation of AMPK and activate the PGC-1α-PPARα pathway remains enigmatic. Another potential target of interest in future studies can be epoxide hydrolase (sEH) as previous studies have shown that inhibiting sEH may be involved in alleviating HFD-induced hepatic adiposity and inflammation (79, 80).

One major limitation of the current study is the number of the examined proteins. Since a large variety of proteins with various functions orchestrates the lipid metabolism process, we could not analyze all these participants. With regard to this, we are planning to utilize proteomics, a powerful tool that characterizes a large scale of proteins by their expressions, functions, structures, and protein-protein interactions (81), in our future studies to acquire a broader perspective. Another limitation is that we only examined liver, although the development of NAFLD could be a joint result of changes in several organs. Therefore, we cannot conclude that carotenoids alleviate NAFLD by directly targeting the liver. Previous studies have reported that carotenoids may mitigate NAFLD *via* the gut- and adipose-liver crosstalk (14). For example, one study showed that disrupted free fatty acid mobilization from mesenteric adipose (MAT) tissue to liver significantly exacerbated NAFLD in mice (82). A potential brain-liver axis involving melanocortin-4 receptor, neuropeptides like neuropeptide Y and agouti-related peptide together regulates food intake, energy expenditure and the pathogenesis of NASH (83, 84). In addition, we failed to provide precise measurement of food consumption of each dietary group as the mice portrayed a behavior of tearing up their food and placing it within their bedding. Although we observed a consistently higher food consumption in the HFD + OC group, compared with other dietary groups, it is difficult to explain whether the orange carrot diet motivated the food intake or promoted physical activity that led the mice to tear more food for bedding. Last but not the least, the β-actin bands were not unanimous in this article. This was due to the different types of gels used. For the high molecular proteins such as MTP, FAS, ACCα, and PGC-1α, we used tris-acetate gels for optimal separation, which impaired the resolution of β-actin. However, the β-actin expressions were identified within each blot, so our statistical analysis was not affected.

## Conclusion

**FIGURE 7:**
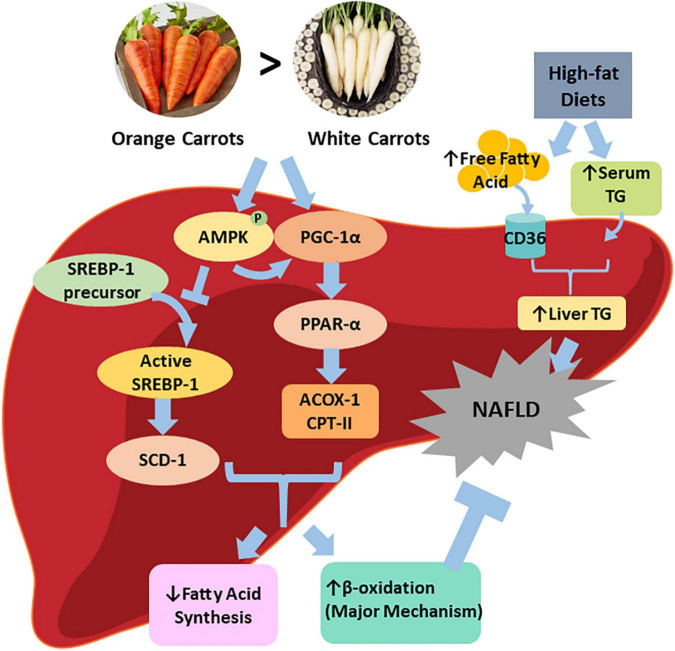
Graphical representation of proposed pathway in which carotenoid-rich orange carrots alleviate NAFLD severity. Orange carrots rich in α-carotene and β-carotene combated the severity of hepatic steatosis brought on by a high-fat diet by partially inhibiting fatty acid synthesis and significantly enhancing β-oxidation proteins, due to promotion of master regulators of hepatic lipid metabolism (i.e., p-AMPK, PGC-1α, PPARα).

Our results showed that orange carrot supplementation was more effective in preventing HFD-induced NAFLD than white carrots, potentially by increasing hepatic β-oxidation through upregulating PPARα (Figure 7). Such data indicate that carotenoid-rich fruits and vegetables may be more efficient in alleviating NAFLD than those with a low carotenoid level. Further clinical trials are warranted to confirm the findings before providing any dietary suggestions to NAFLD patients.

## Data availability statement

The original contributions presented in this study are included in the article/Supplementary material, further inquiries can be directed to the corresponding author.

## Ethics statement

The study was approved by the Institutional Animal Care and Use Committee (IACUC) at North Carolina State University (protocol code #21-004 and date of approval: 02/26/2021).

## Author contributions

AE: supervision, project administration, and funding acquisition. All authors conceptualization, methodology, software, validation, formal analysis, investigation, resources, data curation, writing—original draft preparation, writing—review and editing, visualization, read, and agreed to the published version of the manuscript.

## Abbreviations

ACC α: acetyl coenzyme A carboxylase alpha
ACOX-1: acyl-CoA oxidase
AMPK: AMP-activated protein kinase
CD36: cluster of differentiation 36
CPT-I: carnitine palmitoyltransferase-I
CPT-II: carnitine palmitoyltransferase-II
DGAT2: diacylglycerol *O*-acyltransferase 2
FAS: fatty acid synthase
HFD: high-fat diet
HFD + OC: high-fat diet w/20% orange carrot powder
HFD + WC: high-fat diet w/20% white carrot powder
IACUC: Institutional Animal Care and Use Committee
LFD: low-fat diet
MTP: microsomal triglyceride transfer protein
NAFLD: non-alcoholic fatty liver disease
PPAR α: peroxisome proliferator-activated receptor alpha
PGC-1 α: peroxisome proliferator-activated receptor gamma coactivator 1-alpha
SCD-1: stearoyl-CoA desaturase-1
SREBP-1: sterol regulatory element-binding protein 1
TG: triglyceride.
