# Supplementary material for: Carotenoids in orange carrots mitigate non-alcoholic fatty liver disease progression
Source: Front Nutr. 2022 Sep 26;9:987103. doi: 10.3389/fnut.2022.987103 (PMC9549209; doi:10.3389/fnut.2022.987103)
Supplement: Supplementary file 1 [file Data_Sheet_1.PDF]

frontiers in Nutrition

SUPPLEMENTAL INFORMATION:

Carotenoids in Orange Carrots Mitigate Non-Alcoholic Fatty Liver Disease Progression

Emilio Balbuena[†1,2], Junrui Cheng[†1] and Abdulkerim Eroglu[*1,2]

[1] Plants for Human Health Institute, North Carolina State University, Kannapolis, NC 28081, USA

[2] Department of Molecular and Structural Biochemistry, College of Agriculture and Life Sciences, North Carolina State University, Raleigh, NC 27607, USA

Carotenoids in Dietary Pellets

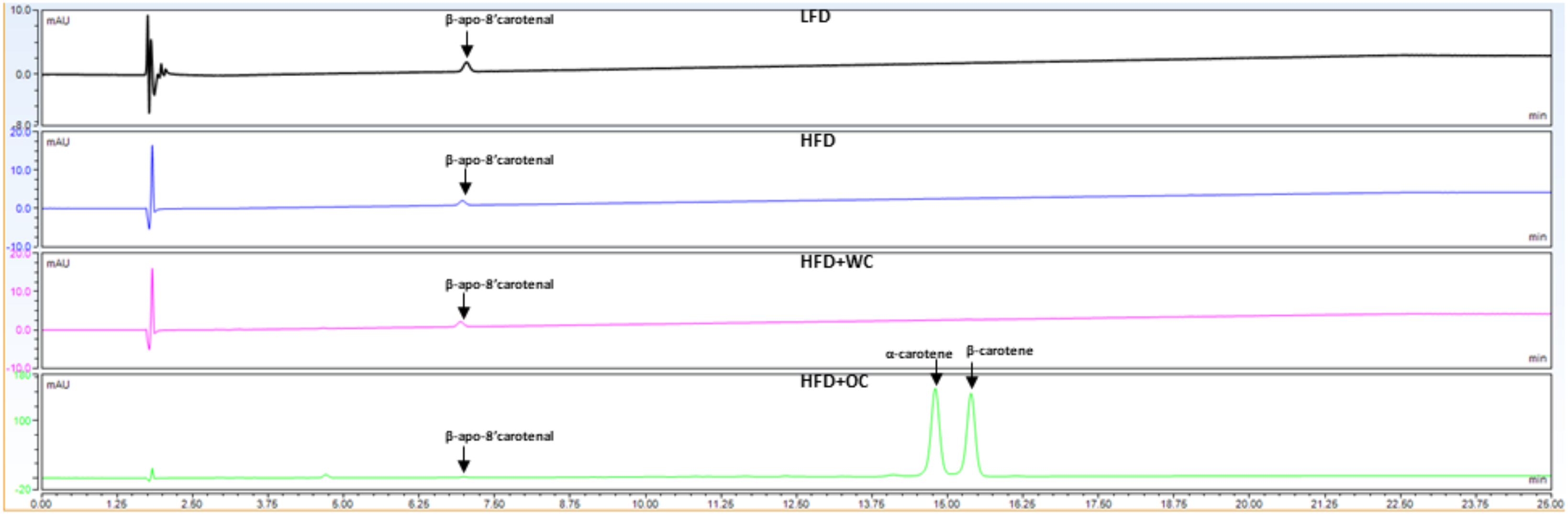

Serum Carotenoids

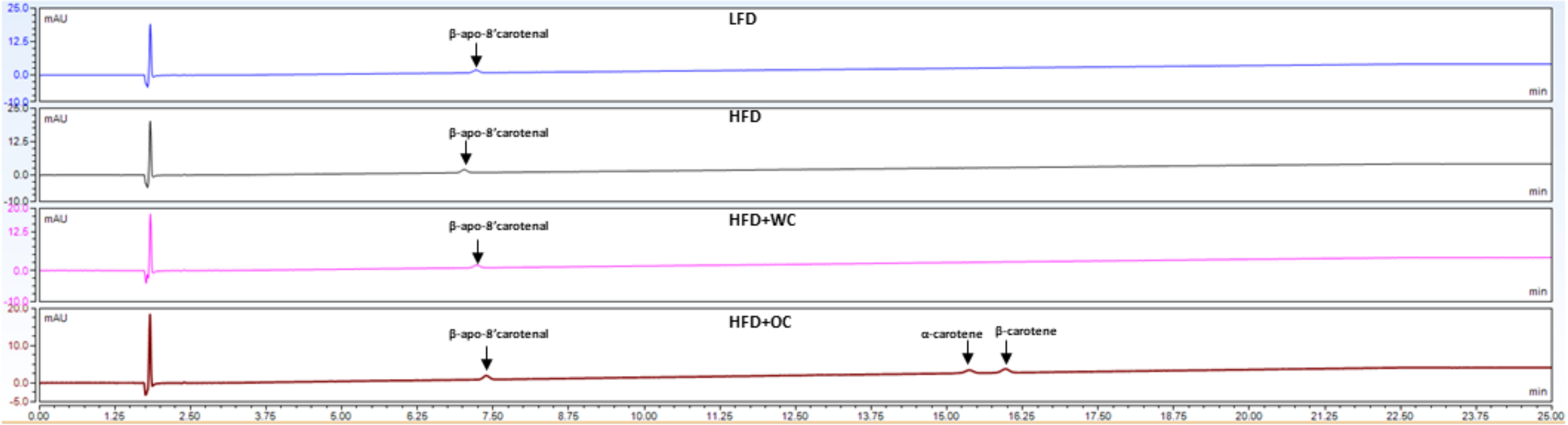

Liver Carotenoids

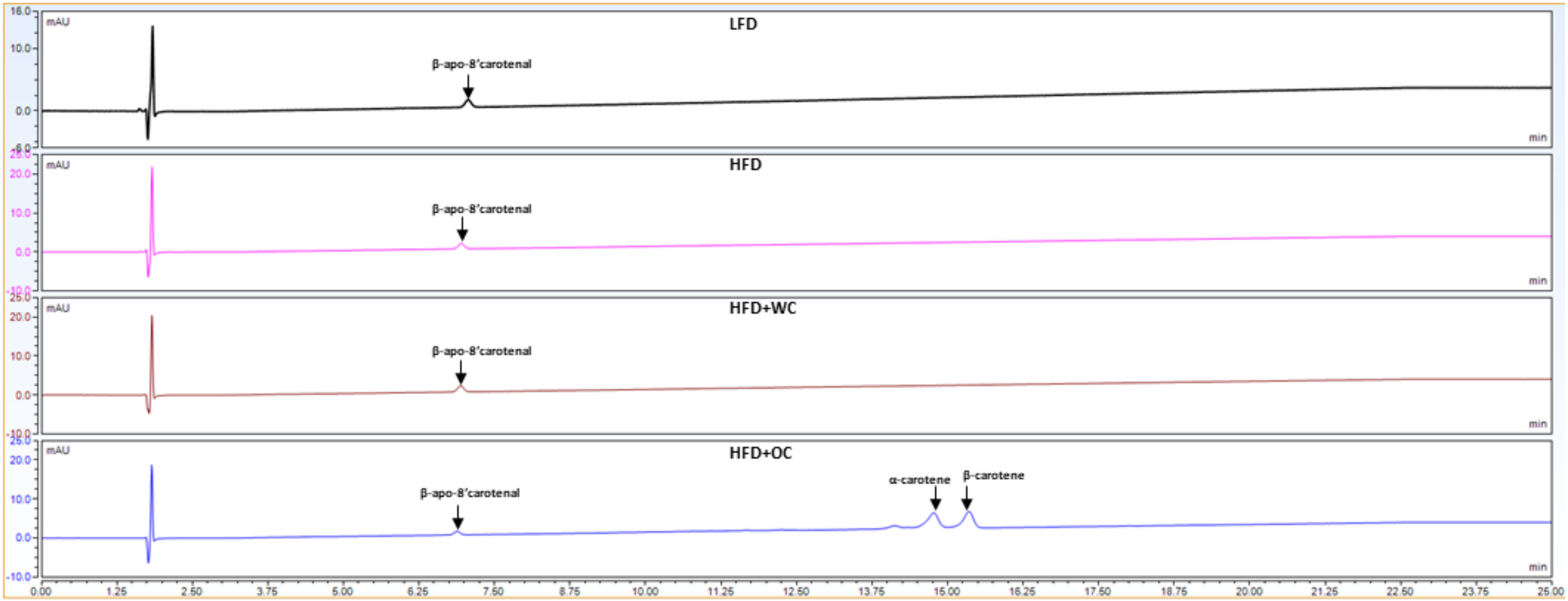

Supplementary Figure 1: HPLC chromatograms of α-carotene and β-carotene in diet pellets, serum, and liver. β-Apo-8'-carotenal used as an internal standard during extractions.

frontiers in Nutrition

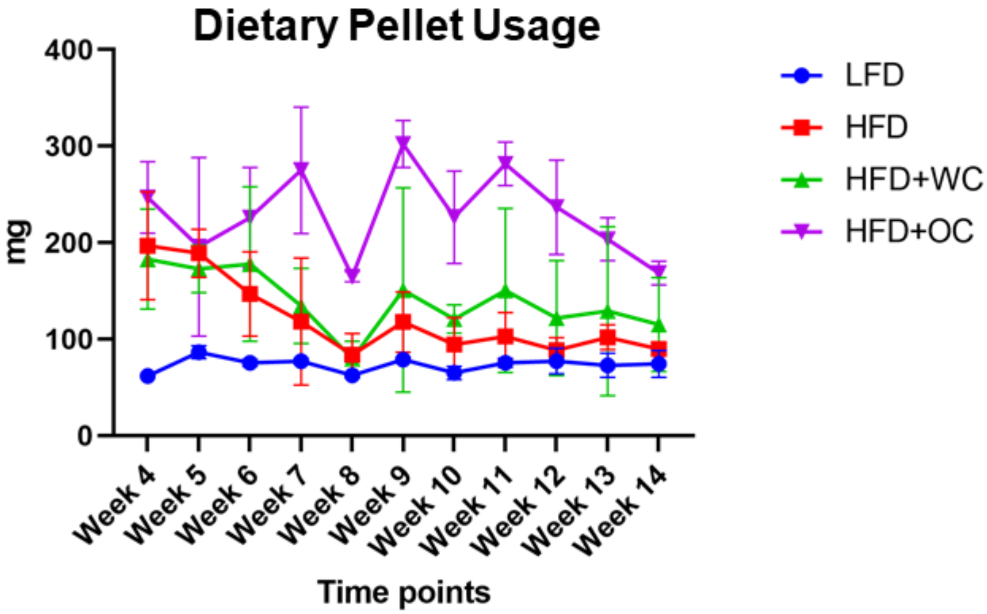

Supplementary Figure 2: Weekly report of dietary pellet loss (consumption and bedding utilization) by weighing food leftover in basket.

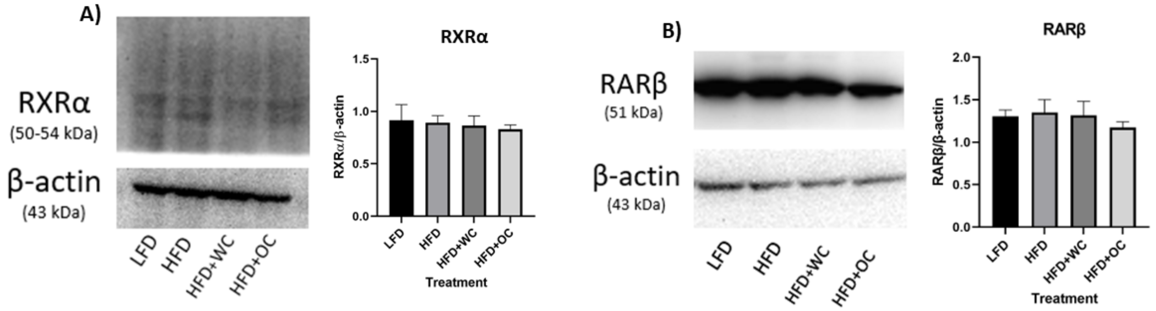

Supplementary Figure 3: Western blots of hepatic **A)** RXRα and B) RARβ. For both western blot, LFD: n=11; HFD, HFD+WC, HFD+OC: n=12.

frontiers
in Nutrition

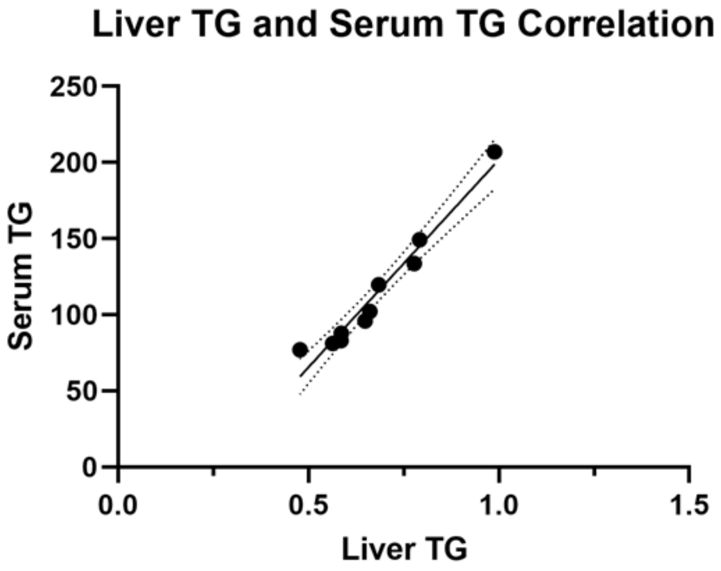

Supplementary Figure 4: Correlation analysis between hepatic and serum triglyceride (TG) content in the HFD group, $p < 0.001$.

A)

ID	D12450J	D12492		
Treatment name	LFD	HFD	White carrot (HFD+WC)	Orange carrot (HFD+OC)
kcal%				
Protein	20	18	19	19
Carbohydrate	70	21	19	19
Fat	10	61	62	62
Ingredient	g	g	g	g
Casein	200	200	200	200
L-Cysteine	3	3	3	3
Corn Starch	506.2	0	0	0
Maltodextrin 10	0	125	20.24	20.24
Lodex	125	0	0	0
Sucrose	72.8	68.8	68.8	68.8
White carrot powder	0	0	154.76	0
Orange carrot powder	0	0	0	154.76
Cellulose, BW200	0	50	0	0
Solka Floc, FCC200	50	0	0	0
Soybean Oil	25	25	25	25
Lard	20	245	245	245
Mineral Mix, S10026	50	10	10	10
DiCalcium Phosphate	0	13	13	13
Calcium Carbonate	0	5.5	5.5	5.5
Potassium Citrate	0	16.5	16.5	16.5
Vitamin Mix, V10001	1	10	10	10
Choline Bitartrate	2	2	2	2
Total (g)	1055.05	773.8	773.8	773.8
kcal/g	3.82	5.11	5.08	5.08

frontiers in Nutrition

B)

Class Description	Ingredient	Grams (g)
Carbohydrate	Sucrose, Fine Granulated	78.42
Vitamin	Vitamin E Acetate, 50%	10.00
Vitamin	Niacin	3.00
Vitamin	Biotin, 1%	2.00
Vitamin	Pantothenic Acid, d. Calcium (B5)	1.00
Vitamin	Vitamin D3, 100,000 IU/g	1.00
Vitamin	Vitamin B12, 0.1% Mannitol	1.00
Vitamin	Vitamin A Acetate, 500,000 IU/g	0.80
Vitamin	Pyridoxine HCl (B6)	0.70
Vitamin	Riboflavin (B2)	0.60
Vitamin	Thiamin (B1)	0.60
Vitamin	Folic Acid	0.20
Vitamin	Menadione Sodium Bisulfite	0.08
		**Total: 100.00 g**

frontiers in Nutrition

Supplementary Table 1: Compositional break down of A) dietary pellets and B) vitamin (V10001) mix formulated by Research Diets, Inc.

Genes:	Forward:	Reverse:
Acox1	TAACTTCCTCACTCGAAGCCA	AGTTCCATGACCCATCTCTGTC
β-Actin	CTTTTCCAGCCTTCCTTCTTGG	CAGCACTGTGTTGGCATAGAGG
Cd36	GCGACATGATTAATGGCACA	CCTGCAAATGTCAGAGGAAA
Cpt	GCACTGCAGCTCGCACATTACAA	CTCAGACAGTACCTCCTTCAGGAAA
Fas	GCTGCGGAAACTTCAGGAAAT	AGAGACGTGTCACTCCTGGACTT
Pparα	GTACCACTACGGAGTTCACGCA	CATTGTGTGACATCCCGACAG
Pgc1α	TATGGAGTGACATAGAGTGTGCT	CCACTTCAATCCACCCAGAAAG
Scd1	CCGGAGACCCCTTAGATCGA	TAGCCTGTAAAAGATTTCTGCAAACC
Srebp1	TAGAGCATATCCCCCAGGTG	GGTACGGGCCACAAGAAGTA

Supplementary Table 2: Primer sequences used in qPCR experiments. Forward and reverse sequences generated with the NCBI BLAST tool.
